# Laxative Effects of Total Diterpenoids Extracted from the Roots of *Euphorbia pekinensis* Are Attributable to Alterations of Aquaporins in the Colon

**DOI:** 10.3390/molecules22030465

**Published:** 2017-03-14

**Authors:** Kuilong Wang, Lian Liu, Jianyu Huang, Hongli Yu, Hao Wu, Yu Duan, Xiaobing Cui, Xingde Zhang, Liping Liu, Wei Wang

**Affiliations:** 1 School of pharmacy, Nanjing University of Chinese Medicine, Nanjing 210023, China; wjwkl@126.com (K.W.); gyhliulian@163.com (L.L.); 18260028133@163.com (J.H.); xiaobingcui@163.com (X.C.); xingde2293@126.com (X.Z.); liuliping1487@163.com (L.L.); zhonglixunta349@163.com (W.W.); 2 Jiangsu Key Laboratory of Chinese Medicine Processing, Nanjing University of Chinese Medicine, Nanjing 210023, China; duanyu1681@sina.com; 3 Engineering Center of State Ministry of Education for Standardization of Chinese Medicine Processing, Nanjing 210023, China; 4 State Key Laboratory Cultivation Base for TCM Quality and Efficacy, Nanjing University of Chinese Medicine, Nanjing 210023, China

**Keywords:** *Euphorbia pekinensis*, diterpenoids, aquaporins, laxative effects

## Abstract

This study was designed to evaluate the toxic effects of total diterpenoids extracted from the roots of *Euphorbia pekinensis* (TDEP) on the mouse colon and to clarify the mechanism. Dried powdered roots of *E. pekinensis* were extracted with chloroform, and then the extract (6.7 g) was subjected to column chromatography and preparative TLC, giving TDEP. Using the HPLC-DAD method, the purity of TDEP was determined as 85.26%. Mice were orally administered with TDEP (3.942, 19.71 and 39.42 mg/kg), after which fecal water content and colon water content were examined. Both of them increased over time after TDEP administration, accompanied by severe diarrhea. Three hours after TDEP administration, the animals were sacrificed to obtain their colons. The mRNA and protein expression levels of aquaporin 1 (AQP1), AQP3 and AQP4 in the colon were measured using real-time RT-PCR and Western blotting, respectively. TDEP significantly increased the levels of AQP3 and AQP4, but decreased that of AQP1 in dose-dependent manners. Similarly, Pekinenin C, a casbane diterpenoid, significantly increased AQP3 protein and mRNA expressions in human intestinal epithelial cells (HT-29). Histopathological examination revealed that the colon was not significantly damaged. The laxative effects of *E. pekinensis* were associated with the alterations of AQPs in the colon by TDEP.

## 1. Introduction

Aquaporins (AQPs), which are water and water/glycerol channels for the rapid transport of water across membranes, are expressed in a variety of tissues in human. So far, 13 types of AQPs have been discovered in mammals [1], and many of them have been proven to play key roles in water transport in the intestinal tract [2,3]. The colon is a place where dehydrated feces are produced. The colon epithelium absorbs about 1.5–2 L/day of water against an osmotic gradient, most of which moves mainly through AQPs. The main AQPs expressed in mucosal epithelial cells in the colon are AQP1, AQP3 and AQP4. Magnesium sulphate, employed as a traditional Chinese medicine for a thousand years, is considered to be an osmotic laxative. However, the latest studies found that the laxative effect of magnesium sulphate is not only caused by changed osmotic pressure in the intestinal tract, but increased AQP3 expression also plays a very important role [4].

*Euphorbia pekinensis*, as a member of the genus *Euphorbia* in the family Euphorbiaceae, has been used to treat gonorrhea, edema, migraines and warts in traditional Chinese medicine [5]. However, it is poisonous and stimulating. When used improperly, it can induce intense diarrhea, abdominal pain, bloody diarrhea and other gastrointestinal toxicities. The main toxic compounds in this plant are diterpenoids; although there are many types of cytotoxic diterpenoids from *Euphorbia pekinensis* [6], the hotspot is casbane-type [7,8,9,10], and they are toxic to many cell lines. Pekinenin C, a casbane diterpenoid separated from *E. pekinensis*, can induce IEC-6 cell apoptosis via both the mitochondrial and death receptor pathways [11].

In this study, we prepared total diterpenoids from *E. pekinensis* (TDEP) through extraction, aiming to investigate the effects on AQPs protein and mRNA expressions in the colon of mice. Besides, we evaluated the effects of Pekinenin C on human intestinal epithelial cells (HT-29) to confirm our results.

## 2. Results and Discussion

### 2.1. Analysis of TDEP

**Figure 1:**
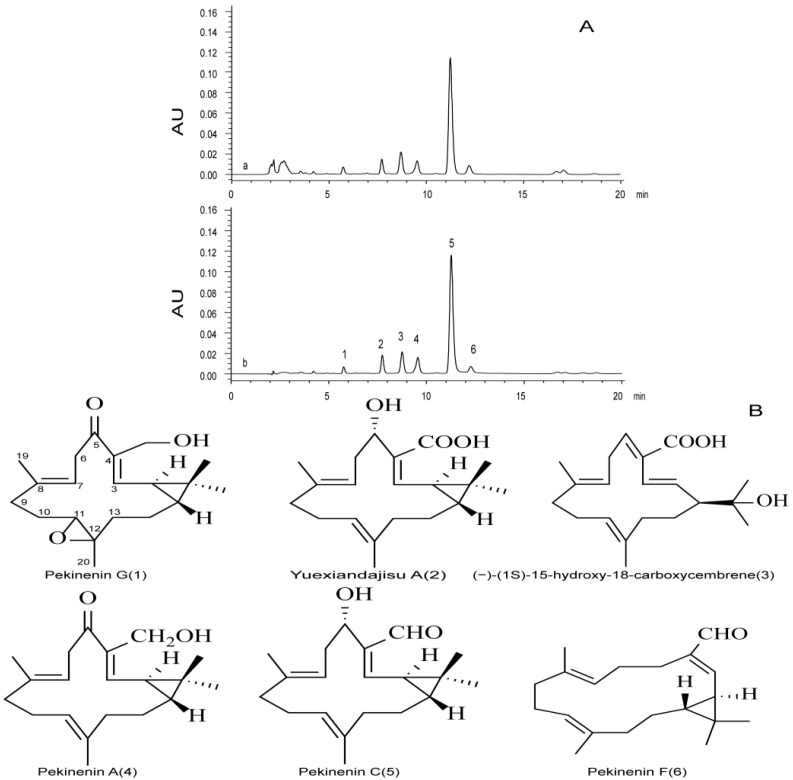
HPLC chromatogram of TDEP (**A**): (a) TDEP, (b) standards; Structure of identified components of TDEP (**B**): six peaks were identified by comparing with the standard substance: Pekinenin G (**1**), Yuexiandajisu A (**2**), (−)-(1S)-15-hydroxy-18-carboxycembrene (**3**), Pekinenin A (**4**), Pekinenin C (**5**), Pekinenin F (**6**).

We obtained six diterpenoids from *E. pekinensis* and used them as standards in this study. TDEP were dissolved in methanol and detected using HPLC. Six compounds were found, accounting for 2.44%, 5.05%, 9.34%, 6.67%, 57.29% and 4.47% of TDEP, respectively (Figure 1).

### 2.2. Effect of TDEP on Fecal and Intestinal Water Contents

**Figure 2:**
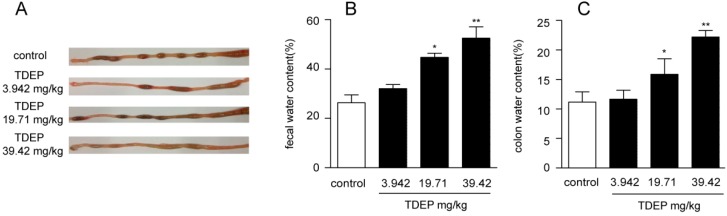
Diarrheal effect of TDEP. Macroscopic evidence of watery stool in the colon of TDEP-treated mice compared to that of control group (**A**); (**B**) TDEP dose-dependently (3.942, 19.71, 39.42 mg/kg) increased the fecal water content compared with the control group mice; (**C**) TDEP dose-dependently (3.942, 19.71, 39.42 mg/kg) increased the colon water content compared with the control group mice. Data are represented as the mean ± SD. * *p* < 0.05, ** *p* < 0.01 vs. control group, respectively. *n* = 6 in each group and each assay was repeated six times.

Feces were collected up to 3 h after oral administration of TDEP for calculation of the fecal water content (Figure 2A). TDEP dose-dependently increased such content of the treated mice, inducing obvious symptoms of diarrhea compared to the normal control group. Similarly, TDEP dose-dependently increased the colon water content (Figure 2).

### 2.3. Histological Results

**Figure 3:**
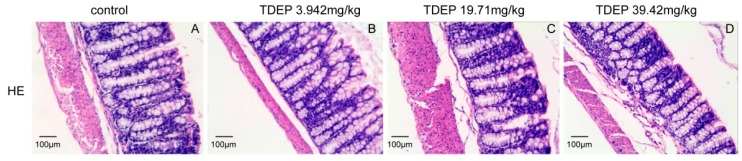
Effect of TDEP on the histological morphology of mouse colon by HE staining. Normal mice (**A**); mice administered with TDEP (3.942 mg/kg) (**B**); mice administered with TDEP (19.71 mg/kg) (**C**); mice administered with TDEP (39.42 mg/kg) (**D**).

Figure 3 shows no significant damage in the colon.

### 2.4. Effects of TDEP on mRNA and Protein Expression Levels of AQPs in the Colon

**Figure 4:**
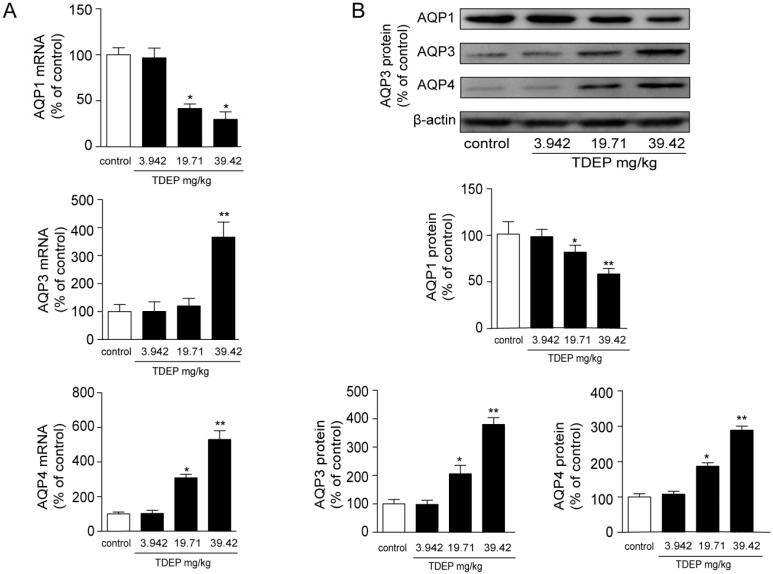
Changes in mRNA expression levels (**A**) and protein expression levels (**B**) of AQPs in the mice colon after the administration of TDEPs; (**A**) Expression level of AQP3 mRNA in mice colons treated by TDEP at different dosages (3.942, 19.71 and 39.42 mg/kg, respectively) for 3 h. The results were normalized with β-actin level and AQPs mRNA level of the control was taken as 100%; (**B**) Expression level of AQPs protein in mice colons treated by TDEP at different dosages (3.942, 19.71 and 39.42 mg/kg, respectively) for 3 h. The results were normalized with β-actin protein level, and all AQPs protein level of the control was taken as 100%. Data are represented as the mean ± SD. * *p* < 0.05, ** *p* < 0.01 vs. control group, respectively. *n* = 3 in each group and each assay was repeated six times.

Figure 4 shows changes in the mRNA and protein expressions of AQPs in the colon after administration of TDEP.

The mRNA expression of AQP3 increased significantly after administration of TDEP, reaching approximately four times that of the baseline level, and that of AQP4 also increased, reaching about four times that of the baseline level. However, the mRNA expression of AQP1 decreased.

The AQPs protein expression levels in the colon were detected by Western blotting. As shown in Figure 4, compared with the normal control group, 3.942–39.42 mg/kg TDEP markedly increased the expressions of AQP3 and AQP4 in a dose-dependent manner, while the expression of AQP1 decreased.

### 2.5. Pekinenin Cand TDEP Increased Gene and Protein Expressions of AQP3 in HT-29 Cells

**Figure 5:**
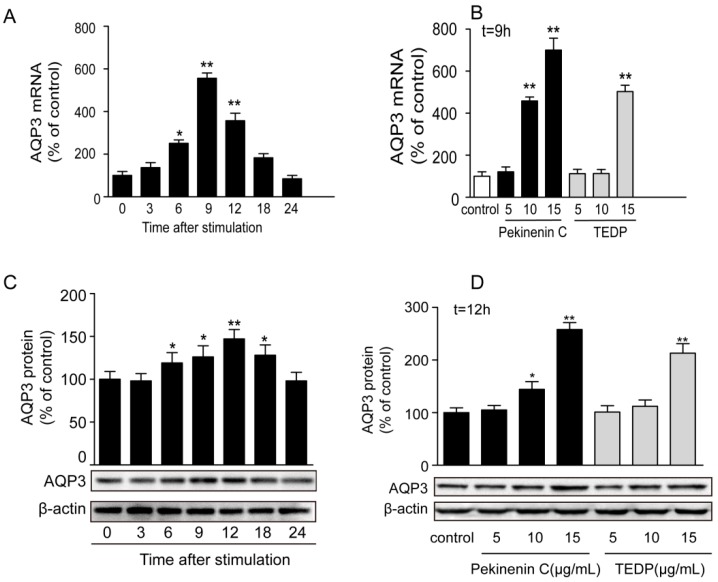
Changes in mRNA expression levels (**A**) and protein expression levels (**B**,**C**) of AQP3 in the HT-29 cells after the administration of Pekinenin C and TDEP; (**A**) Expression level of AQP3 mRNA intreated by Pekinenin C (15 μg/mL) for different times (0, 3, 6, 9, 12, 18 and 24 h, respectively); (**B**) Expression level of AQP3 mRNA in HT-29 cells treated by Pekinenin C and TDEP at different dosages (5, 10 and 15 μg/mL, respectively) for 3 h. The results were normalized with β-actin level, and AQPs mRNA level of the control was taken as 100%; (**C**) Expression level of AQP3 mRNA intreated by Pekinenin C (15 μg/mL) for different times (0, 3, 6, 9, 12, 18 and 24 h, respectively); (**D**) Expression level of AQP3 protein in HT-29 cells treated by Pekinenin C and TDEP at different dosages (5, 10 and 15 μg/mL, respectively) for 12 h. The results were normalized with β-actin protein level, and AQP3 protein level of the control was taken as 100%. Data were represented as the mean ± SD. * *p* < 0.05, ** *p* < 0.01 vs. control group, respectively. *n* = 3 in each group and each assay was repeated six times.

In this study, Pekinenin C and TDEP were added to HT-29 cells. This cell line has been widely used to examine the effects on the AQP3 protein expression level, as well as to investigate the mechanisms of diarrhea development. Figure 5 shows the changes of mRNA and protein expressions after Pekinenin C and TDEP administrations.

Firstly, the mRNA expression of AQP3 significantly increased and peaked 9 h after the administration of Pekinenin C, reaching three times that of the baseline level (0 h), and then decreased with prolonged time. As exhibited in Figure 5A, 9 h of administration with Pekinenin C and TDEP markedly increase the mRNA expression level of AQP3 in HT-29 cells dose-dependently.

Secondly, the Western blot results showed that after Pekinenin C (15 μg/mL) administration, the AQP3 protein expression level increased significantly and peaked at 12 h, and was almost twice that of the baseline level. After 12 h of treatment, Pekinenin C and TDEP increased AQP3 protein expression dose-dependently.

## 3. Discussion

*E. pekinensis* has been widely used to treat gonorrhea, edema, migraines and warts in traditional Chinese medicine, but improper use can induce intense diarrhea and other gastrointestinal toxicities [6]. Casbane-type diterpenoids are the toxic compounds in this plant. The casbane diterpenoid from *E. pekinensis* has been reported to induce IEC-6 cell apoptosis [11]. However, most studies only paid attention to the effect of the casbane diterpenoid on cell lines in vitro and its action in vivo, with the toxicity effect hitherto unknown. In this study, we prepared TDEP according to the properties of diterpenoids, using Pekinenin C as a reference. The purity of TDEP was detected by HPLC; however, only six kinds of diterpenoids were obtained and used as standards, which accounted for 85.26% TDEP, and the remaining 14.74% could be other diterpenoids which we did not obtain. According to this, the TDEP extract could replace the total diterpenoids. Mice were orally administrated with different doses of TDEP, leading to sever colon edema 3 h later, and wet stool was collected. TDEP increased both the feces water content and colon water content in a dose-dependent manner. Moreover, Pekinenin C, a casbane diterpenoid, accounted for 57.29% of TDEP. Its toxicity was further confirmed in HT-29 cells, indicating that casbane diterpenoids were the main toxic compounds in *E. pekinensis* by causing edema in the colon and diarrhea.

The intestinal mucosal barrier consists of four parts: a mechanical barrier, a chemical barrier, a biological barrier and an immune barrier [12]. The mechanical barrier is closely related to water transport. Intestinal water moves through both the paracellular route and the transcellular route. For a long time, the paracellular route has been considered to predominantly control water movement. Nevertheless, the transcellular route has now been regarded as mainly transporting water in the colon due to rigid tight junctions in the epithelial cells [13,14]. AQPs, as water channels, have been involved in water transport in the intestinal tract [15], playing critical roles in the transcellular route in the colon. So far, 13 types of AQPs have been discovered in mammals [16]. AQPs that are mainly expressed in mucosal epithelial cells in the colon are AQP3 and AQP4 [17,18], and AQP1 is mostly expressed in microvessels. In this study, TDEP obviously increased both the mRNA and protein expressions of AQP3 and AQP4 in mucosal epithelial cells in the colon, but decreased AQP1 expression.

**Figure 6:**
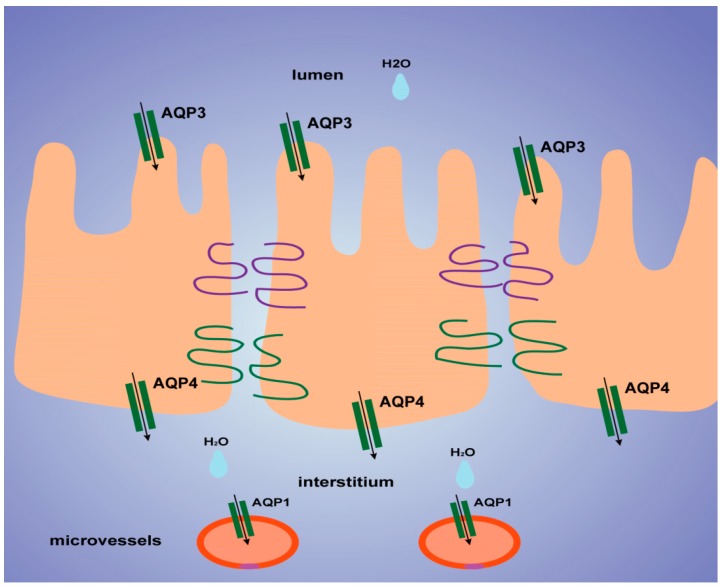
A possible transcellular route (aquaporins, AQPs) of water movement across the gastrointestinal tract epithelia. In the colon, water moves both in the absorption and in the secretion direction. AQP3 are localized at the apical side, and AQP4 at the basolateral side. Water is transported into or out of the mucosal microvessels through AQP1.

Histological results showed that the colon was not significantly damaged, suggesting that the mechanical barrier of the colon remained untouched. Therefore, the laxative effect of TDEP cannot be ascribed to damage to the paracellular route. However, given severe edema in the colon and the laxative effect, the transcellular route may be responsible, probably owing to alterations of AQPs in the colon. Herein, we detected the mRNA and protein levels of AQP1, AQP3 and AQP4 in mouse colon. Both the mRNA and protein levels of AQP3 and AQP4 increased, whereas those of AQP1 decreased. Similar to previous literature [19,20], we found that AQP3 was expressed mostly on the luminal side of the colon mucosa, AQP4 was expressed mostly in the submucosal layer of the colon, and AQP1 was expressed mostly in microvessels, as shown in Figure 6. Thus, we tentatively clarified the mechanism by which TDEP induced diarrhea and colon edema in mice. Firstly, AQP3 and AQP4 increased, which promoted water movement from the lumen to the colon interstitium. Nevertheless, AQP1 expression decreased, so water in the colon interstitium could not be transferred through microvessels. As a result, sever edema occurred in the colon, finally resulting in the laxative effect due to accumulated water.

The toxic effect of TDEP was further confirmed in HT-29 cells, using Pekinenin C as a standard. Both of them increased AQP3 expression in HT-29 cells, but the mRNA expression of AQP3 in Pekinenin C–treated HT-29 cells exceeded that of TDEP-treated ones. Also, Pekinenin C elevated AQP3 protein expression more significantly, because TDEP contained 14.74% of compounds other than diterpenoids. In addition, the in vitro experiments suggested that diterpenoids were indeed the main toxic compounds in *E. pekinensis.*

In conclusion, for the first time, we attributed the laxative effect of *E. pekinensis* mainly to casbane-type diterpenoids, and the toxic effects were associated with alterations of AQPs in the colon. Particular attention should be paid to herbal medicines that contain casbane-type diterpenoids, such as *Euphorbia ebracteolata* [21] and *Euphorbia hylonoma* and *Euphorbia wangii* [22] which also belong to the genus *Euphorbia.* Given the crucial roles of AQPs [23], it is promising to develop new laxatives and antidiarrheal agents targeting them in the intestine.

## 4. Experimental Section

### 4.1. Preparation of TDEP

The toxic compounds in *E. pekinensis* are diterpenoids, most of which are casbane-type, being soluble in low-polarity organic solvents such as chloroform, acetone and ethyl acetate. Also, the diterpenoids can be detected by vanillin–sulphuric acid to confirm the preparation process of TDEP.

Dried powdered roots (100 g) of *E. pekinensis* were extracted three times with 100 mL of chloroform, 30 min each time. After filtration and removal of the solvent under reduced pressure, the chloroform extract (6.7 g) was subjected to column chromatography (silica gel, gradient of PE–EtOAc), affording fractions 1–2 (PE–EtOAc 30:1, 10:1), and fraction 2 was subjected to preparative TLC and developed with PE–EtOAc (2:1). Pekinenin C was used as a reference, and spot was detected by spraying with vanillin–sulphuric acid, followed by heating, finally yielding a yellow oil (876 mg).

### 4.2. Determination of TDEP

Six diterpenoids, i.e., pekinenin G, pekinenin F, Yuexiandajisu A, pekinenin A, and pekinenin C, were separated from *E. pekinensis* as reported [8], and dissolved in methanol into appropriate concentrations. TDEP were also dissolved in methanol to detect the chemical compositions using HPLC.

HPLC analysis was performed on a Waters 2695 separations module system equipped with a photodiode array detector (Waters, Milford, MA, USA), Agilent C18 column (250 mm × 4.6 mm, 5.0 mm), an analytical column of reverse-phase HPLC, was eluted gradiently with a mobile phase of A (water and 0.1% formic acid) and B (methanol) (75%–85% B from 0 to 20 min). The flow rate of the mobile phase was 1 mL·min^−1^, and the column temperature was maintained at 30 °C. The injection volume was 30 μL, and the column eluents that directly flowed into the photodiode array detector were detected at 268 nm. 

### 4.3. Animals and Treatment

Twenty-four male ICR mice (18–22 g) were obtained from JOINN Laboratories, Suzhou, China (License No.: SCXK (SU) 2013-0003). They were kept in a temperature-controlled environment (22 ± 2 °C) with 55% ± 5% relative humidity and a 12 h/12 h light/dark cycle, and fed with standard chow for at least 1 week before any manipulations. The present study was conducted in accordance with the Guiding Principles for the Care and Use of Laboratory Animals, as adopted by the Committee of Animal Research at Nanjing University of Chinese Medicine.

The mice were fasted for 12 h before the yellow oil administration (water provided ad libitum). They were divided into 4 groups randomly with equal numbers (*n* = 6): a normal control group and three yellow oil (3.942, 19.71, 39.42 mg/kg, respectively)-treated groups. The four groups were orally administrated by syringe-feeding with distilled water (0.3 mL/kg) or the yellow oil. Three hours after administration, the colons were removed. After the intestinal tract was washed with 9% NaCl solution, some portions were fixed in 10% formalin in phosphate-buffered saline, dehydrated, and then embedded in paraffin for histopathological examination. The other portions were flash-frozen with liquid nitrogen and stored at −80 °C.

### 4.4. Feces Water Content

Feces samples from the mice were collected for up to 3 h after administration of the yellow oil and placed in silica gel, followed by drying for 24 h in a desiccator. Water content per gram of feces was calculated based on the difference between wet and dry fecal weights. These percentages were averaged among six animals for each group. 

### 4.5. Colon Water Content

Mice were sacrificed 3 h after administration of TDEP, and the colons were removed. After being washed with PBS and patted dry on a filter paper, the wet colons were weighed. Afterwards, the colons were placed in an oven at 60 °C for 4 h. Water content per gram of colon was calculated based on the difference between wet and dry colon weights

### 4.6. Histopathological Examination

The colon samples (*n* = 5) were fixed in 10% phosphate-buffered formalin, dehydrated and then embedded with paraffin. Sections of about 4 μm thickness were sliced from each embedded tissue and stained with hematoxylin-eosin (HE). The sections were observed under a light microscope with magnification of 400× by a pathologist blinded to the experimental profile.

### 4.7. Cell Culture

Human colon cancer HT-29 cells were purchased from the Type Culture Collection of the Chinese Academy of Sciences (Shanghai, China). They were cultured in RPMI 1640 medium supplemented with 10% FBS, 100 U/mL penicillin G potassium and 100 μg/mL streptomycin at 37 °C, which were inoculated on a six-well plate at 1 × 10^6^ cells/cm^2^, incubated in a CO_2_ incubator at 37 °C for 12 h, and then treated with the yellow oil dissolved in culture medium for 3 h. The cells were collected using a cell scraper, and the expressions of AQP3 RNA and protein were measured by real-time RT-PCR and Western blotting. Experiments were done using cell lines that had previously been passaged five to 15 times.

### 4.8. RNA Preparation and SYBR Green Real-Time RT-PCR

Total RNA from colons of mice and HT-29 cells was isolated using Trizol reagent. The resulting solution was diluted 50-fold using DEPC water, and the purity and concentration (µg/mL) of RNA were calculated by measuring the absorbances at 260 and 280 nm using a BioPhotometer plus spectrophotometer (Eppendorf, Hamburg, Germany). A high-capacity cDNA synthesis kit (Thermo, Waltham, MA, USA) was used to synthesize cDNA from 1 μg of RNA according to the instruction manual. The expressions of target genes were detected by preparing the primers listed in Table 1 and by performing real-time RT-PCR. Each well of a 96-well PCR plate was added 10 µL of SYBR green super mix, 0.2 µL of forward primer of the target gene (10 pmol/µL), 0.2 µL of reverse primer (10 pmol/µL), 2 µL of cDNA TE buffer solution, and 6.8 µL of RNase-free water

### 4.9. Preparation of Tissue and Cell Extraction for Western Blot

Colon tissues were removed and immediately soaked in ice-cold, oxygenated phosphate-buffered saline. Then 50 mg tissue was carefully cleaned and 500 µL of lysis buffer was added. The tissue was then homogenized using a digital homogenizer (Bioprep-24, Shanghai, China) on ice. The tissue homogenate was centrifuged (16,000× *g*, 4 °C for 5 min), and the supernatant was stored at –80 °C until use.

After stimulation for 3 h, the culture supernatant was discarded, and HT-29 cells were washed with PBS buffer. Then the cells were carefully scraped off on ice and centrifuged at 2000 rpm for 5 min. After the supernatant was discarded, the residue was added cytomembranelysis solution, left in ice bath for 5 min, and centrifuged at 14,000 rpm for 10 min to collect the supernatant rich in cytoplasmic protein.

### 4.10. Electrophoresis and Immunoblotting

The protein concentrations were measured using the BCA method with BSA as a standard. Electrophoresis was performed using the Laemmli’s method. The protein (6 μg) was diluted two-fold using loading buffer (84 mMTris, 20% glycerol, 0.004% bromophenol blue, 4.6% sodium dodecyl sulfate, and 10% 2-mercaptoethanol; pH 6.8) and applied to a 12% polyacrylamide gel. After electrophoresis, the isolated proteins were transferred to a polyvinylidene difluoride membrane. After blocking for 2 h using 1% skimmed milk, the resulting membrane was reacted for 2 h at room temperature with goat anti-rabbit AQP1 (EnoGene, Nanjing, China), AQP3 (Abcam, Cambridge, UK), AQP4 (EnoGene, Nanjing, China) antibody (1/500 for mouse colon; 1/100 for HT-29 cells) respectively. After washing by TBS-Tween (20 mM Tris–HCl, 137 mM NaCl and 0.1% Tween 20, pH 7.6), the resulting membrane was reacted for 1 h at room temperature with horse radish peroxidase-labeled anti-rabbit IgG antibody (Invitrogen Corp., Carlsbad, CA, USA 1:2000). After washing, the membrane was reacted with ECL Plus detection reagent and visualized with LAS-4000 mini (FujiFilm, Tokyo, Japan), a Luminoimage analyzer.

### 4.11. Statistical Analysis

SPSS version 16.0 for Windows (SPSS, Chicago, IL, USA) was used for statistical analysis. Numerical data were expressed as mean ± SD. The significance of differences was examined using analysis of variance, followed by the Dunnett’s test. Results with *p* < 0.05 were considered statistically significant.

## Figures and Tables

**Table 1:** Primer sequences of mRNA.

Gene	Forward (5—3)	Reverse (5—3)	Amplicon Size (bp)
Mouse AQP1	CTGCTGGCGATTGACTACAC	TCCACACCTTCATGCGGTC	190
Mouse AQP3	GTGATGTTTGGCTGTGGCTC	TTCAAGTGGGCACCAGACAC	184
Mouse AQP4	AAGGCGGTGGGGTAAGTG	TGAGCCACCCCAGTTTATGG	187
Mouse β-actin	GACCCAGATCATGTTTGAGAC	GTAGCCACGCTCGGTCAG	168
Human AQP3	CTGGTGGTCCTGGTCATTG	CACATTCTCTTCCTCGTTGG	163
Human β-actin	GACCCAGATCATGTTTGAGAC	GTAGCCACGCTCGGTCAG	179

## Supplementary Materials

Supplementary materials are available online.
