# Supplementary material for: Laxative Effects of Total Diterpenoids Extracted from the Roots of Euphorbia pekinensis Are Attributable to Alterations of Aquaporins in the Colon
Source: Molecules. 2017 Mar 14;22(3):465. doi: 10.3390/molecules22030465 (PMC6155307; doi:10.3390/molecules22030465)
Supplement: Supplementary file 1 [file molecules-22-00465-s001.pdf]

The mass spec profile for each peak. As you know, there are too many Isomers in diterpenoids, and the response of diterpenoids is not satisfied in UHPLC-ESI-MS. We detect 1-5 compounds, but Pekinenin F is not detected using this method, so we also provide the NMR date of the six compounds.

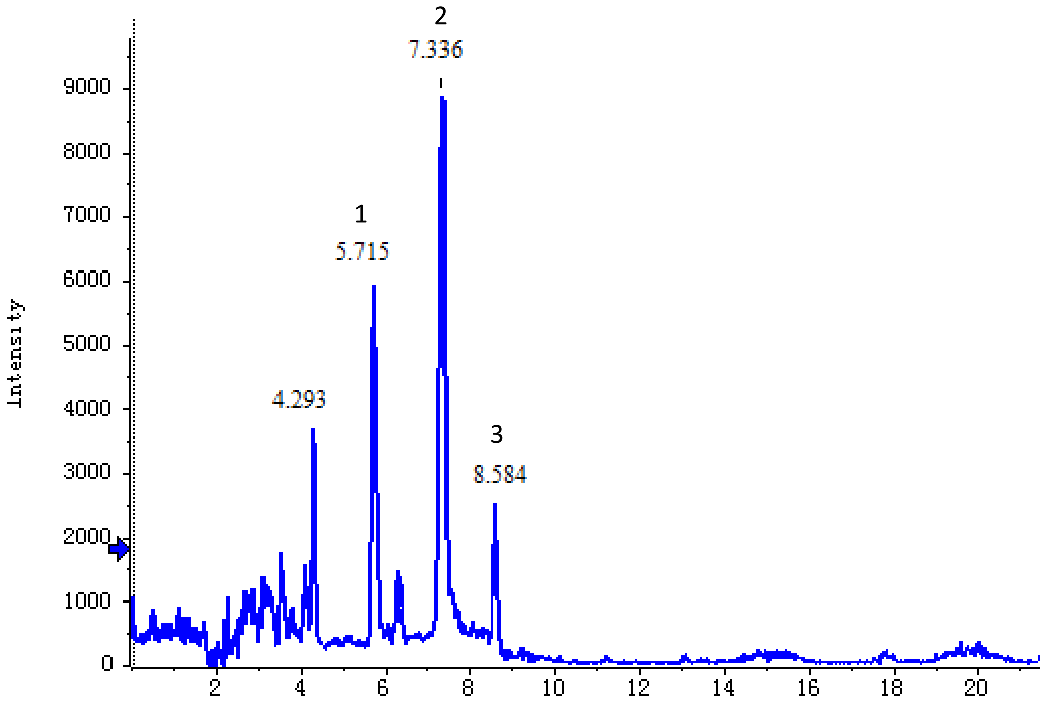

Figure S1. Mass spec profile for Pekinenin G (1), Yuexiandajisu A (2), (−)-(1S)-15-hydroxy-18-carboxycembrene (3)

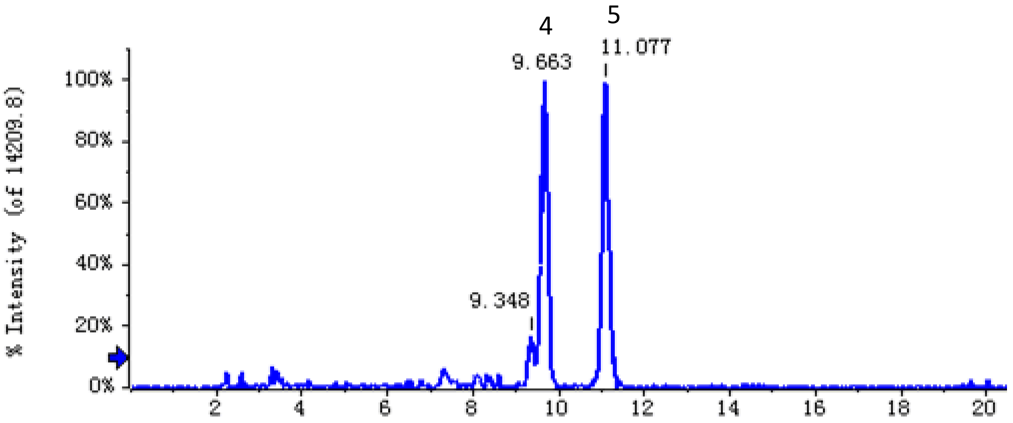

Figure S2. Mass spec profile for pekinenin A (4), pekinenin C (5)

Table S1

T_R/min	$[M-H]^-$	Fragment ions	Formula	Mass Error (ppm)	Purity Score
4.293, 5.715, 7.336, 8.584	317	317,273,180, 136,112,68	$C_{20}H_{30}O_3$	-2.6	94.7%
9.663, 11.077	301	301,164,120, 81	$C_{20}H_{30}O_2$	-2.6	87%

Pekinenin G

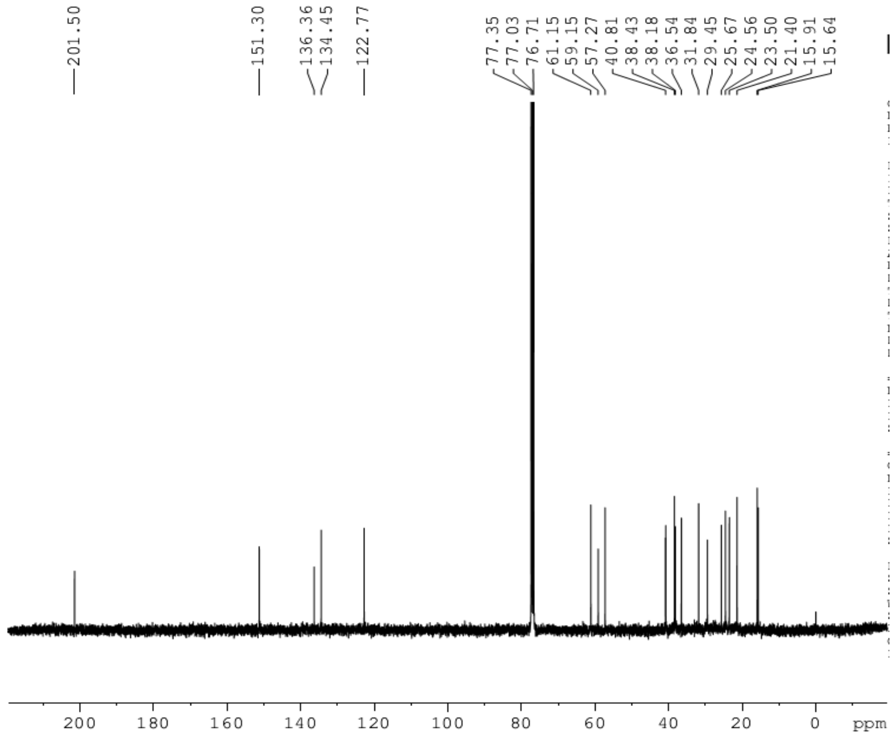

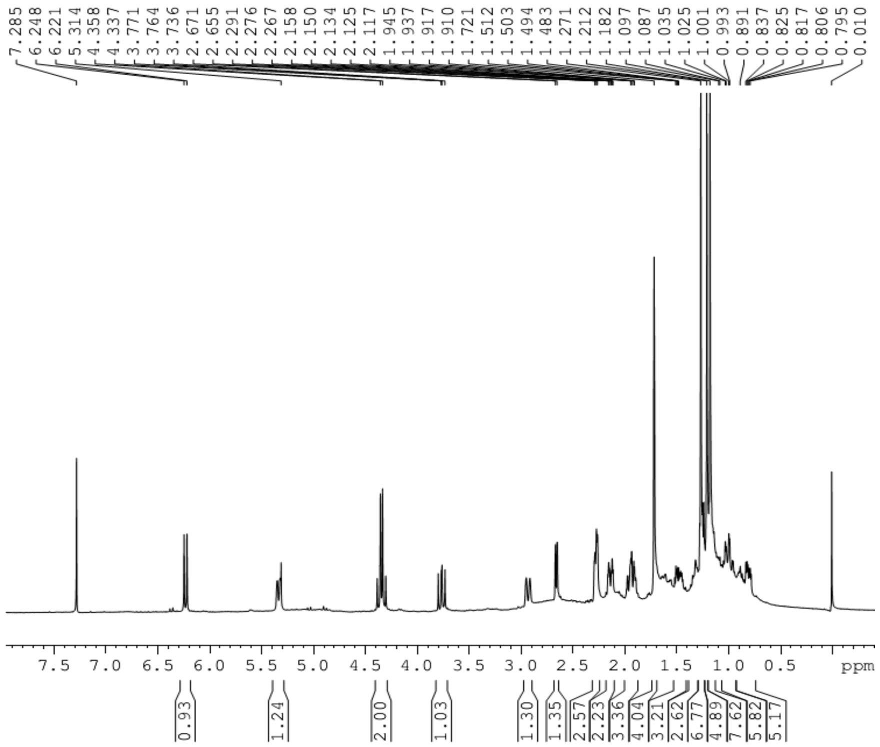

Reference

Wang, K.; Yu, H.; Wu, H.; Wang, X.; Pan, Y.; Chen, Y.; Liu, L.; Jin, Y.; Zhang, C. A new casbane diterpene from *Euphorbia pekinensis*. *Nat. Prod. Res.* **2015**, *29*, 1456–1460.

Yuexiandajisu A

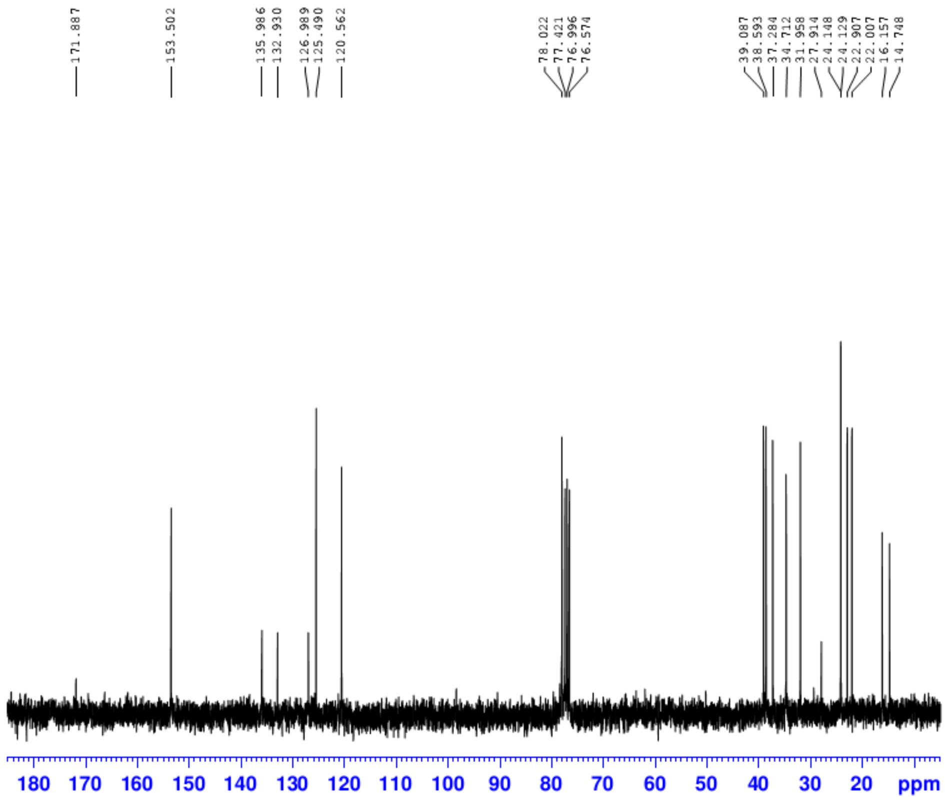

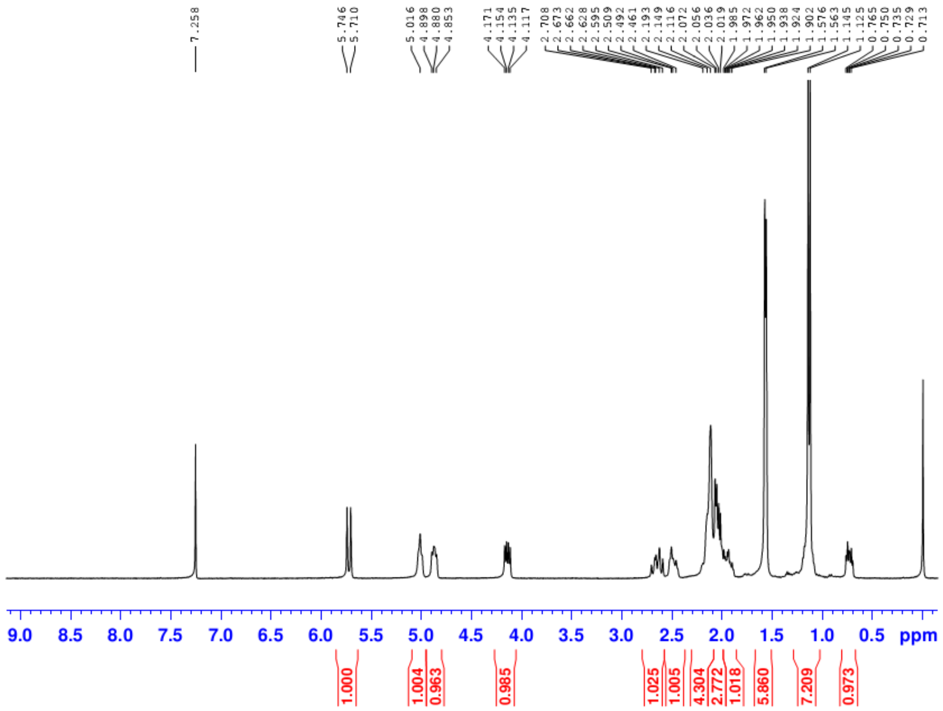

Reference

Zhihong Xu, Jie Sun, Rensheng Xu, Guowei Qin, Casbane diterprnoids from *Euphorbia ebracteolata*. Phytochemistry. 1998,49:149-151.

(−)-(1S)-15-hydroxy-18-carboxycembrene

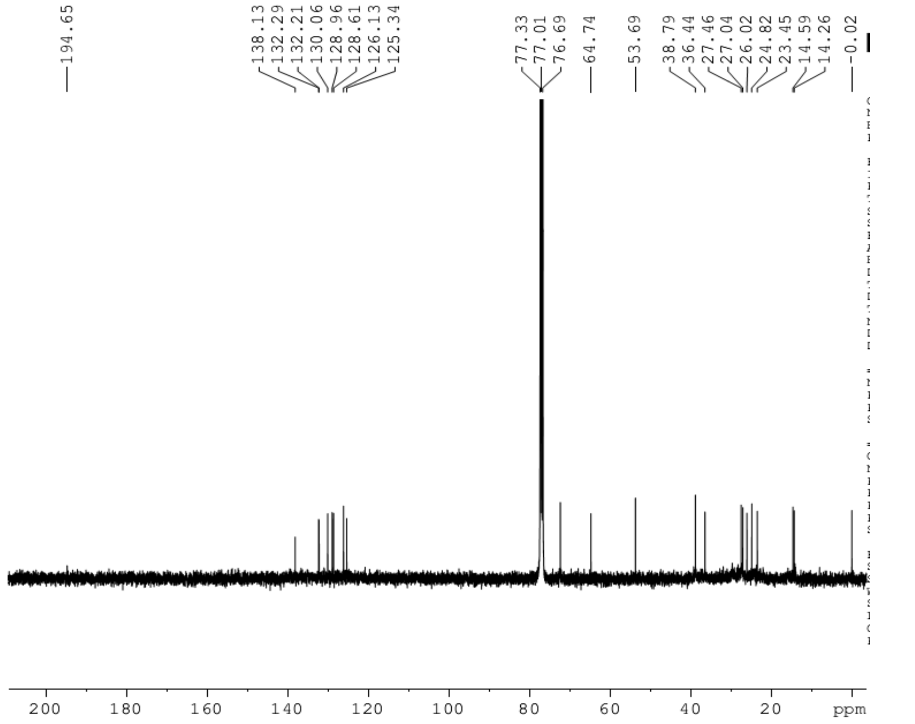

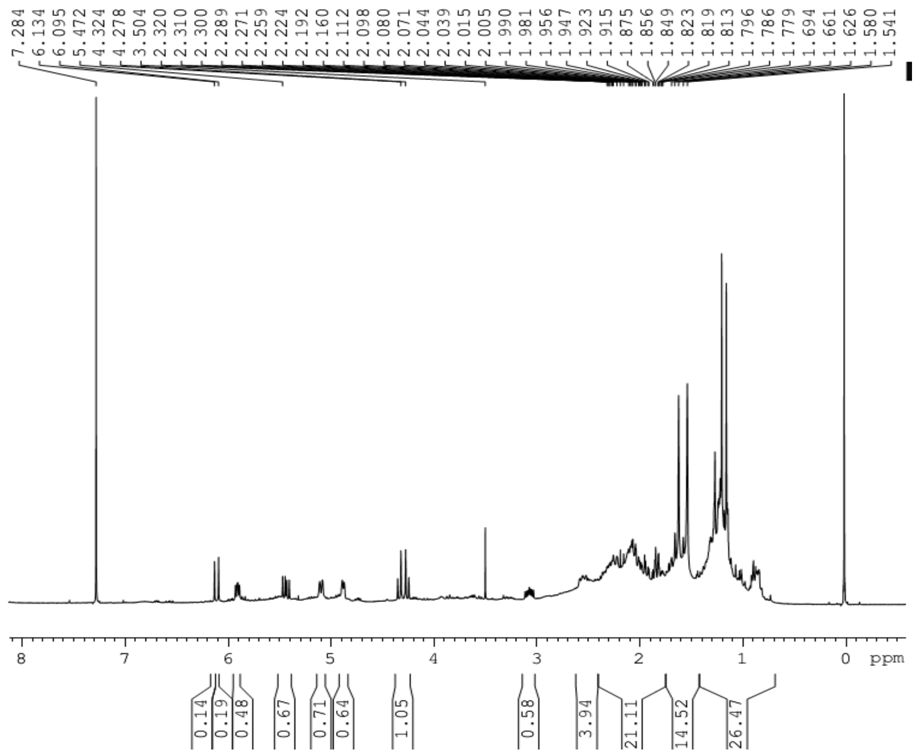

Reference

Pengyi Hou, Yan Zeng, Bingjie Ma, Kaishun Bi, Xiaohui Chen, A new cytotoxic cembrane diterpene from the roots of *Euphorbia pekinensis* Rupr, Fitoterapia, 2013,90:10-13

Pekinenin A

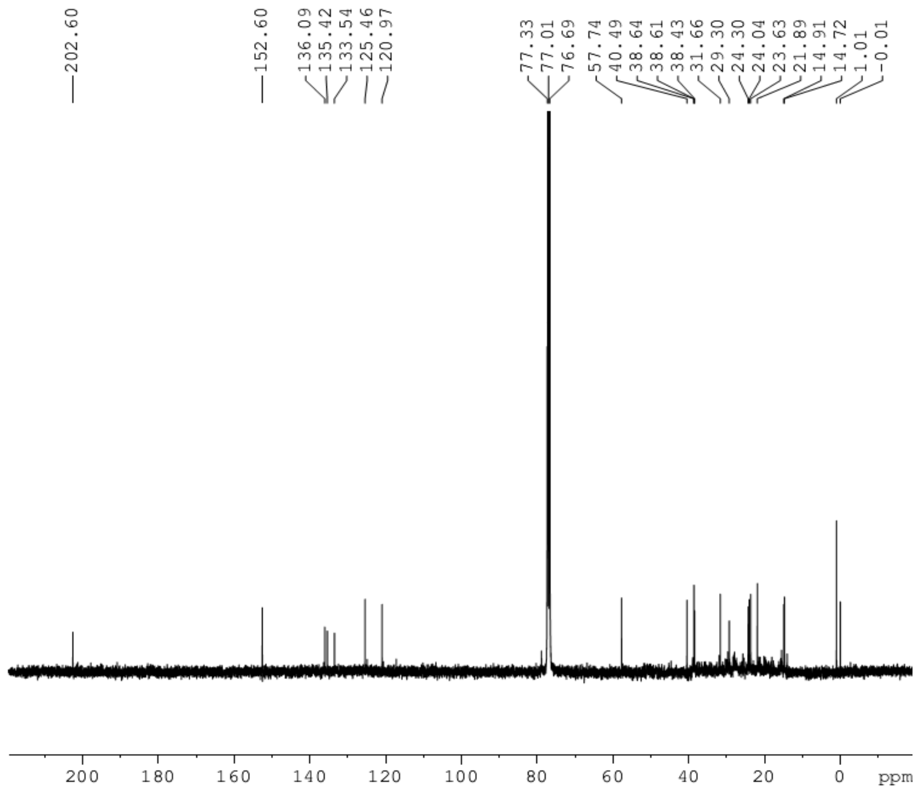

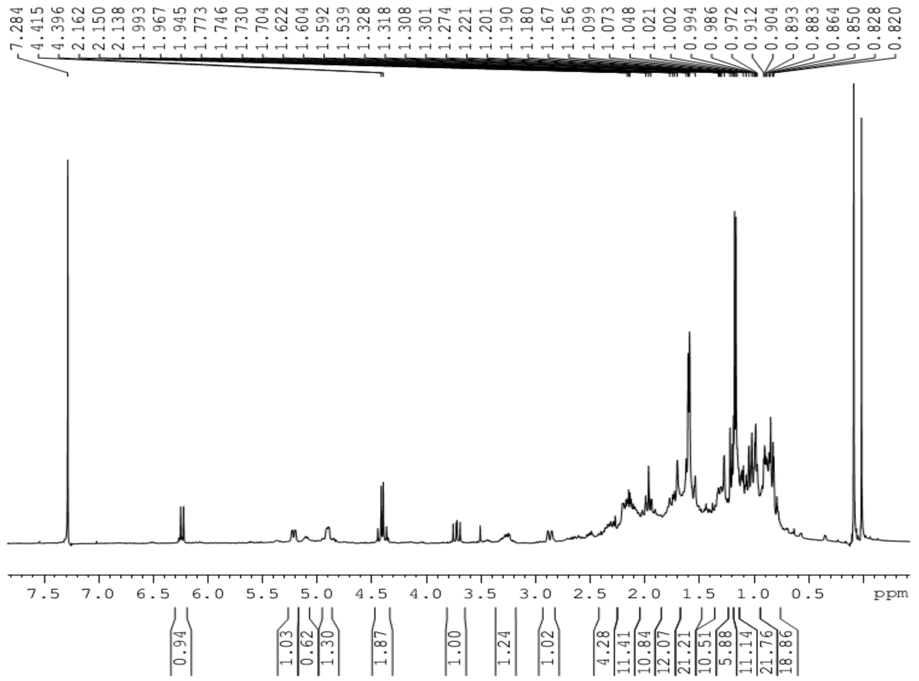

Reference

Shao, F.; Bu, R.; Zhang, C.; Chen, C.; Huang, J.; Wang, J. Two new casbane diterpenoids from the roots of *Euphorbia pekinensis*. *J. Asian Nat. Prod. Res.* **2011**, *13*, 805–810.

Pekinenin C

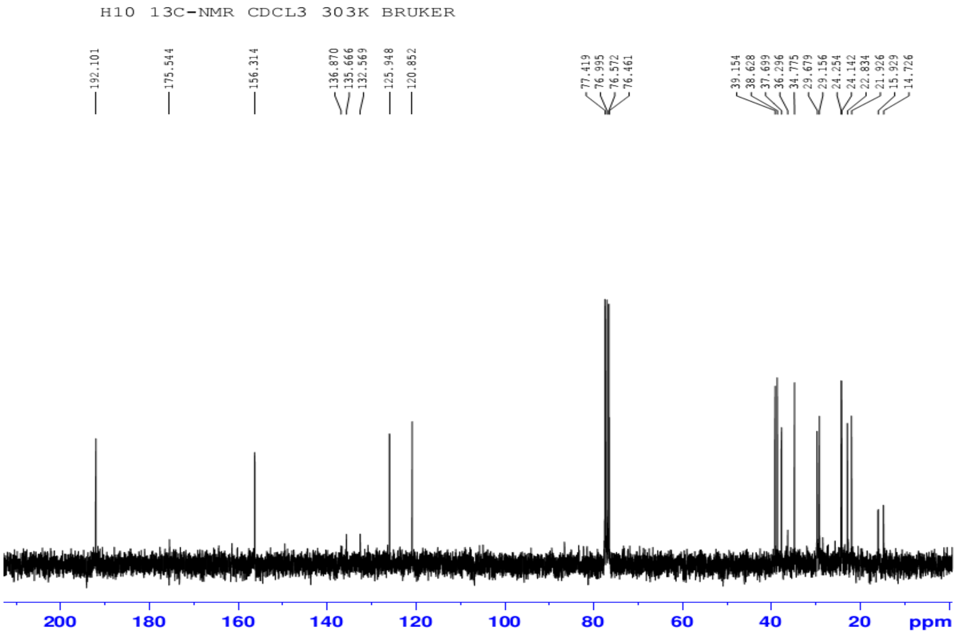

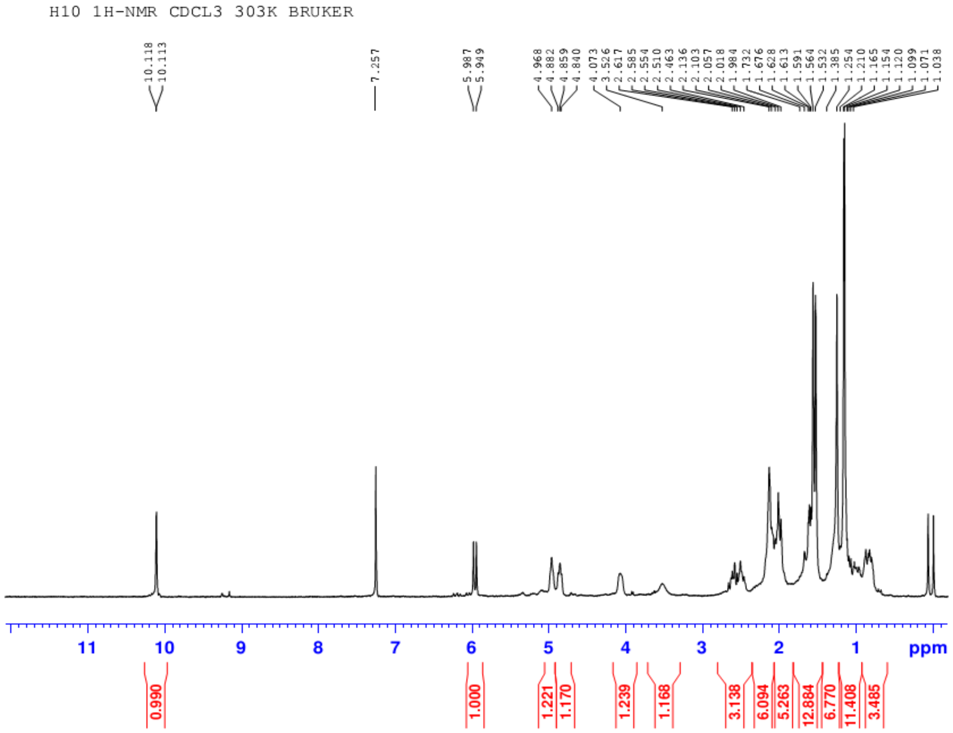

Reference

Tao, W.; Duan, J.; Tang, Y.; Yang, N.; Li, J.; Qian, Y. Casbane diterpenoids from the roots of *Euphorbia pekinensis*. *Phytochemistry* **2013**, *94*, 249–253.

Pekinenin F

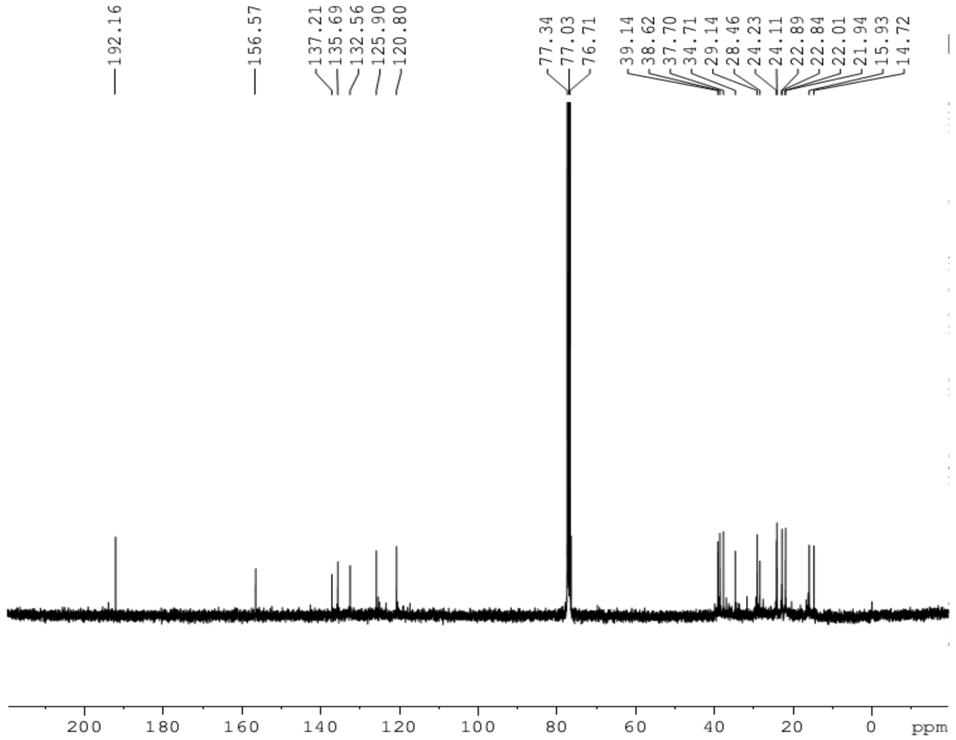

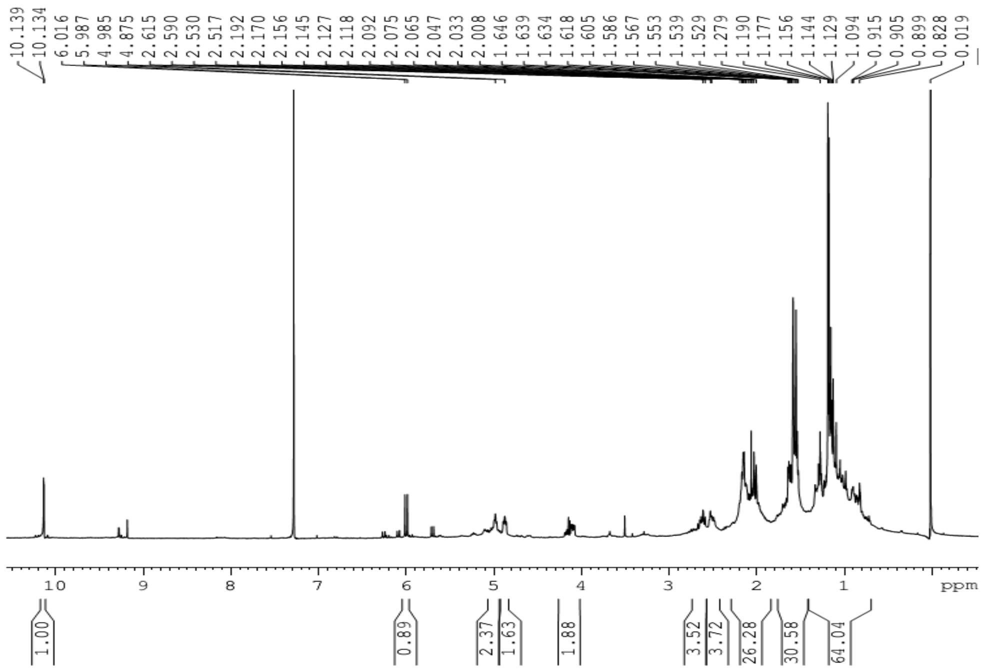

Reference

Tao, W.; Duan, J.; Tang, Y.; Yang, N.; Li, J.; Qian, Y. Casbane diterpenoids from the roots of *Euphorbia pekinensis*. *Phytochemistry* **2013**, *94*, 249–253.
